# Pilomatricoma in a Patient With Chronic Low Testosterone: A Case Report and Literature Review

**DOI:** 10.7759/cureus.77859

**Published:** 2025-01-22

**Authors:** Brittany Tran, Jason Vayner, Joel Jacob, Gina McNew

**Affiliations:** 1 Internal Medicine, Arkansas College of Osteopathic Medicine, Fort Smith, USA; 2 Internal Medicine, Conway Regional Hospital, Conway, USA

**Keywords:** benign skin tumor, calcifying epithelioma of malherbe, hair follicle matrix, low testosterone, pilomatricoma, pilomatrixoma, soft tissue masses

## Abstract

Pilomatricoma is a benign skin tumor with a variety of presentations, making it difficult to recognize. Its rarity further complicates diagnosis, as it is often mistaken for a lipoma or an epidermal inclusion cyst. Although its etiology remains unclear, early identification is crucial due to the risk of malignant transformation into pilomatrix carcinoma. Here, we report the case of a 41-year-old male with a large pilomatricoma on the upper right arm, emphasizing the possibility of this rare tumor arising in an uncommon demographic.

## Introduction

Pilomatricoma, also referred to as pilomatrixoma or calcifying epithelioma of Malherbe, is a rare benign tumor originating from the hair follicle matrix and is most commonly found on the face, scalp, and upper extremities. It is classified into four stages: early, fully developed, early regressive, and late regressive [1]. Although the exact pathophysiology remains unclear, studies indicate that a mutation in the CTNNB1 gene is observed in approximately 75% of cases [2]. This gene encodes the β-catenin protein, which plays a key role in cellular adhesion and communication within the Wnt pathway. In the hair follicle matrix, β-catenin supports hair shaft growth, but when continuously activated, it leads to uncontrolled cell division, contributing to the formation of pilomatricoma.

Although extremely rare, with only 130 cases reported, pilomatricoma can undergo malignant transformation into pilomatrix carcinoma, an aggressive skin cancer capable of metastasizing to the lymphatic system and lungs [3]. Pilomatricoma has also been linked to myotonic dystrophy, familial adenomatous polyposis-related syndromes, Turner syndrome, and Rubinstein-Taybi syndrome. Consequently, patients presenting with six or more pilomatricomas are advised to undergo further screenings for underlying conditions [4].

Pilomatricomas represent less than 1% of all benign skin tumors and are typically painless. They often present as firm subcutaneous nodules, most frequently in pediatric populations and more commonly in females [1,5]. Due to its rarity and diverse presentations, clinically diagnosing this benign tumor can be challenging. We recently encountered a case of pilomatricoma in a 41-year-old male, which is an atypical age and demographic for its onset. In this report, we explore unusual features of the tumor and its potential associations with low testosterone, tattoos, and intramuscular and subcutaneous injections.

## Case presentation

A 41-year-old male with a medical history of hypertension, chronic low testosterone managed with subcutaneous testosterone cypionate, anxiety, and sleep apnea presented to his primary care clinic in October 2024 with bilateral, mobile arm masses enclosed subcutaneously. The mass on the right was located anterolaterally on the upper arm, with an estimated diameter of 2.0 cm. It had grown over the past six months but stopped prior to excision. The mass on the left was located laterally on the upper arm, with an estimated diameter of 1.0 cm, and had not increased in size since its emergence. The patient reported that the masses did not cause pain with direct pressure but caused some irritation if a large force was applied in a vertical direction. No prior imaging or biopsy had been performed on the masses, as the patient had not been concerned. Abnormal laboratory values from a prior visit are summarized in Table 1. However, since the values were only slightly abnormal, the patient was not considered to have any organ dysfunctions or medication side effects prior to the emergence of the masses.

**Table 1:** Abnormal lab values from March 2024 prior to the emergence of the masses Lab values not shown in the table were unremarkable. The testosterone value reflects the trough level prior to the subcutaneous testosterone injection. BUN, blood urea nitrogen; GFR, glomerular filtration rate; LDL, low-density lipoprotein

Test	Patient’s value	Normal value
BUN	20 mg/dL	7-18 mg/dL
GFR	87.0 mL/min	>90 mL/min
Glucose	106 mg/dL	70-100 mg/dL
LDL	126 mg/dL	10-100 mg/dL
Testosterone	251 ng/dL	300-950 ng/dL
BMI	33.6 kg/m²	18.5-24.9 kg/m²

**Figure 1:**
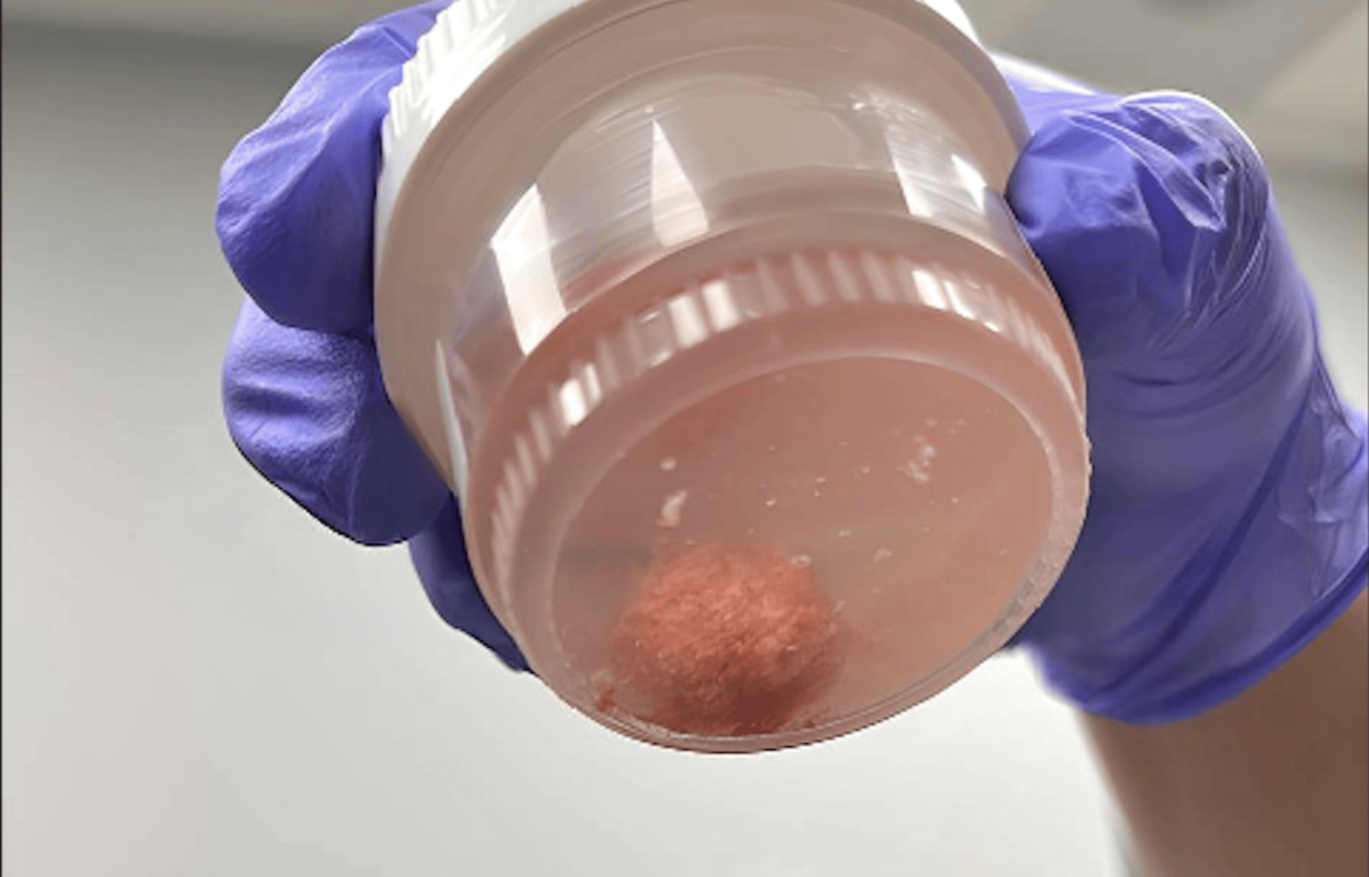
Excisional mass taken from the upper right arm

Physical examination revealed mobile, subcutaneous masses. Although painless, the patient sought removal of the mass in his right arm for comfort and cosmetic reasons. Following betadine sterilization, 10 mL of 1% lidocaine was injected as local anesthesia around the mass, and a small 3.0 cm vertical incision was made for removal. The excised mass, shown in Figure 1, was placed in a collection cup containing formalin and sent for further testing. It was measured at 2.0 g, with dimensions of 2.0 × 1.7 × 1.3 cm. Sectioning of the mass revealed yellow-tan to white-tan firm tissue with a chalky cut surface.

**Figure 2:**
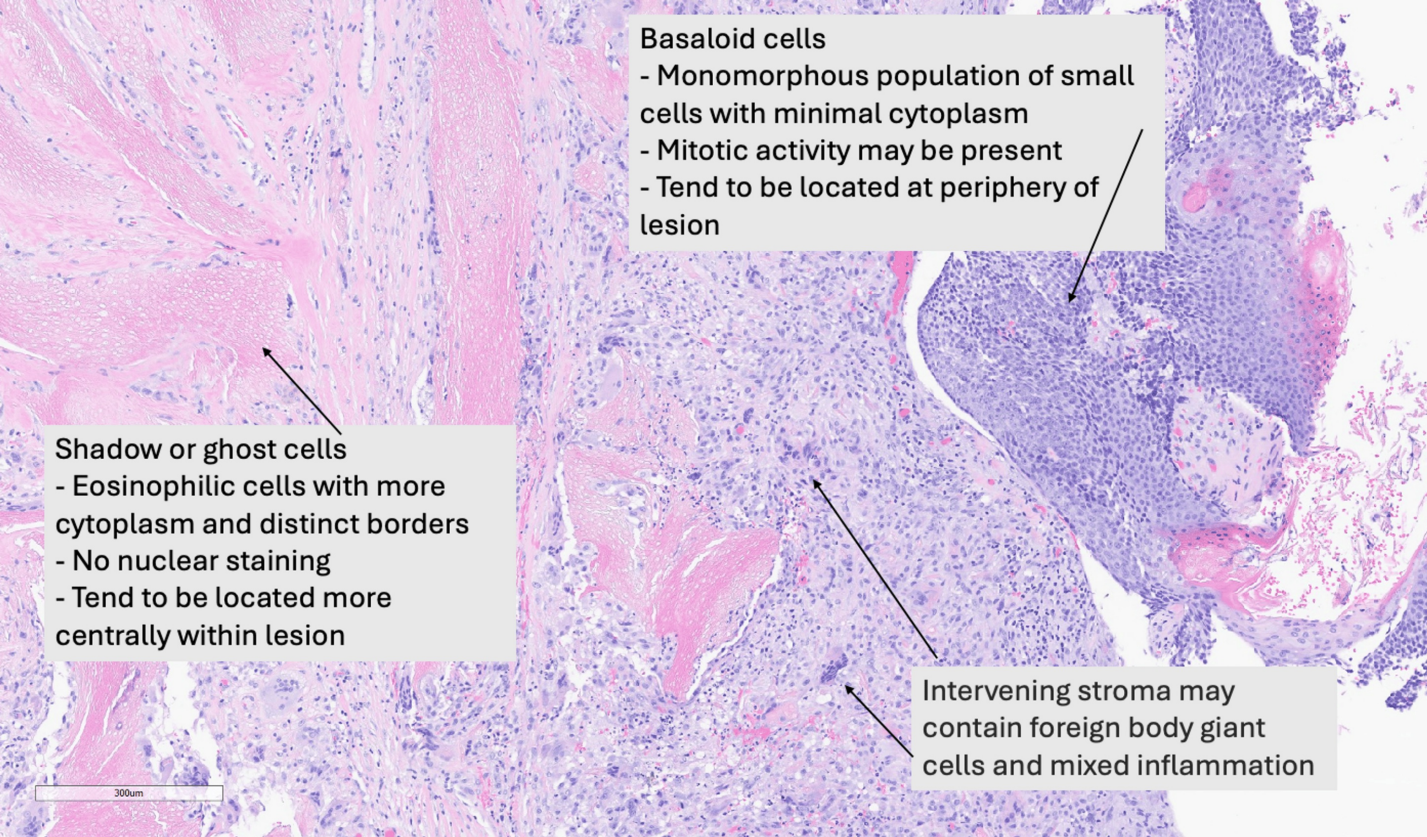
Pathology stain showing the features of pilomatricoma

Histological sections, shown in Figure 2, revealed that the excised mass contained basaloid cells, which were a monomorphous population of small cells with minimal cytoplasm located on the periphery of the lesion. Shadow cells, also known as ghost cells, were present centrally in the lesion. These cells consisted of eosinophilic material without nuclei, resulting in a negative nuclear stain. The presence of these two cell types was indicative of a tumor arising from the hair follicle matrix. As a result, the excised mass was determined to be pilomatricoma with no malignant features. The patient was informed of the diagnosis and was recommended to undergo complete excision of the mass on the left arm for proper preventive treatment and to avoid tumor progression. 

## Discussion

Benign skin tumors are a common concern presented to both primary care providers and dermatologists. While some may be malignant, the majority are benign with many being diagnosed as cysts or lipomas. Pilomatricomas exhibit a bimodal distribution, with peaks during the first and sixth decades of life. About 90% of cases occur in individuals aged five to 15 years, while the remaining cases are seen in those aged 50 to 65 years [5,6]. These tumors are also more commonly observed in females, with a 2:1 female-to-male ratio [5]. Our case, however, presents pilomatricoma in a male patient, and at an age that deviates from the typical bimodal distribution. Additionally, the mass appeared as a skin-colored subcutaneous nodule without any erosions breaking through the skin surface, differing from the characteristic bluish discoloration often reported in other cases [5]. The clinical features of pilomatricoma can vary widely between patients, which often leads to misdiagnosis. Therefore, it is crucial to understand the potential etiologies, characteristics, and presentations of this benign tumor to prevent misdiagnosis and inappropriate treatments.

In addition to the role of the β-catenin protein in the hair follicle matrix, which promotes the growth of the hair shaft, estrogen also plays a key role in hair follicle development. Estrogen binds to high-affinity estrogen receptors on hair follicles, which extends the anagen phase of the hair cycle for thicker and longer hair [7]. While no direct correlation has been established, the higher prevalence of pilomatricoma in women may be related to their increased estrogen levels compared to men. This trend could explain the typical presentation of pilomatricoma in the younger female population, where estrogen levels surge during puberty (typically between nine and 13 years of age), as well as the bimodal distribution seen in postmenopausal women (typically between 48 and 55 years of age), who are more likely to take estrogen supplements to manage postmenopausal symptoms [8]. In our patient, chronic low testosterone levels may have contributed to the development of pilomatricoma due to frequent subcutaneous testosterone injections. Testosterone is converted to estrogen via the aromatase enzyme, and the increased testosterone levels from replacement therapy may result in elevated estrogen levels. Moreover, the aromatase enzyme in men is found in adipose tissue, and since our patient is classified as having class I obesity, there is an expected increase in this enzyme, potentially leading to higher estrogen levels. This altered estrogen-to-testosterone ratio, relative to other men, could explain the presence of this benign tumor in a demographic outside the usual trend. However, since a direct link between low testosterone or elevated estrogen levels and pilomatricoma development has not been established, further research is needed to confirm the etiology.

In this patient’s case, black tattoo ink was present at the site of the benign tumor. Although tattoos have been associated with a variety of other cutaneous conditions, such as dermatomyofibroma, epidermoid inclusion cysts, lipomas, and pilomatricomas, their correlation with these tumors has only been reported in one case each [9]. Therefore, until further studies are conducted, it cannot be definitively concluded that the patient’s pilomatricoma developed as a result of his tattoos. Another potential external factor linked to the development of this benign tumor is the use of intramuscular and subcutaneous injections. For example, a prior study reported a large pilomatricoma associated with daily diclofenac intramuscular injections [10]. While our patient had received multiple corticosteroid injections in his upper arms for joint pain, the specific injection site in relation to the tumor location remains unclear, making it difficult to establish a direct correlation between the injections and the pilomatricoma. Additionally, subcutaneous testosterone cypionate was injected weekly to address his chronic low testosterone levels. Although the exact injection sites are unknown, typical subcutaneous injection areas include the abdomen, thighs, and upper arms - the same location where the benign tumor was found. The repeated trauma to the tissues from intramuscular and subcutaneous injections may explain the presence of inflammation and lymphocytes in the histological analysis of the excised mass. Moreover, this etiology could account for the unusually large size of the tumor, which was twice the size of those typically reported.

As with most other benign skin tumors, the primary treatment for pilomatricoma is surgical excision, as these tumors do not typically regress on their own. Although the lumps are generally painless, the optimal timing for removal remains unclear and may depend on factors such as the patient’s age, comfort, diagnostic concerns, or cosmetic preferences [11]. While surgical removal is not considered an emergency procedure, it is still recommended due to the rare but possible risk of malignant transformation. Additionally, early excision of the benign tumor has been associated with improved scar healing outcomes [11].

Our case presents a unique instance of pilomatricoma, marked by unusual factors such as the age of onset, size, and presentation. Although the exact etiology remains unclear, recurrence of pilomatricoma after excision is rare; when it does occur, it warrants investigation for pilomatrix carcinoma. Fortunately, routine follow-up visits allow for monitoring of the excision sites and the early detection of any benign tumors that may arise in new areas. The aim of this case report was to highlight the distinctive presentation of pilomatricoma in our patient and explore the possible etiologies contributing to its development. While rare, increased research and awareness of pilomatricoma would help the medical community better understand its underlying causes, as well as its symptoms and clinical presentations, which are crucial for early diagnosis and the prevention of malignant transformation.

## Conclusions

This case highlights an atypical presentation of pilomatricoma in a male patient, whose age of onset deviates from the typical pattern. The patient presented with bilateral masses on the upper arms, one of which was confirmed to be a benign tumor measuring 2.0 cm in diameter. Both masses were skin-colored, subcutaneous, and lacked punctures or erosions. Surgical excision remains the primary treatment for pilomatricoma, and further testing of the excised mass provides a definitive diagnosis. The purpose of this case study was to report these rare findings and discuss potential associations contributing to the tumor's formation, such as low testosterone levels, skin irritation from tattoos, and repetitive intramuscular and subcutaneous injections. Although pilomatricoma accounts for less than 1% of all benign skin tumors, it is important to consider it as part of the differential diagnosis, ensuring that accurate treatment and follow-up care are provided.
